# Annotations

**Published:** 1906-06-30

**Authors:** 


### ANNOTATIONS.

### Protective Vaccination against Tuberculosis.

Complete immunity against tuberculosis is probably comparatively rare, yet in the majority of people the tubercle bacillus can only gain a temporary foothold. Most of us seem unable to resist the invasion of the bacillus, but we rapidly acquire protection against its ravages. At least such would appear to be the teaching of the post-mortem room, where slight thickening or other evidence of quiescent tuberculosis at the apex of one or both lungs is one of the commonest of appearances. There are forces within us that are able to fight this as they are able to fight so many other diseases, and upon several lines attempts have been made to aid the body in the conflict. Calmette seems to think that immunity may be acquired by a method of vaccination. Tubercle bacilli killed by heat given by the mouth to a calf are said to protect against infection with active bacilli. If further observations should confirm those already made we must suppose that although a certain degree of heat kills the bacilli and prevents further growth, it does not destroy important chemical constituents of the bacilli. These constituents, when they find entrance to the body, have the power to stimulate it to form products which prevent the growth of living tubercle bacilli. Curiously enough, these observations of Calmette's raise another question. If those are right who believe that bovine tuberculosis is communicable to man, then boiling cow's milk in order to destroy tubercle bacilli would, according to Calmette, not destroy tuberculous substances that have the power to affect the body. Tubercle bacilli, however, are, when present in milk, not then in sufficient numbers to provide poisons that can be expected to do much harm.

### For Young People in Lodgings.

A correspondent of "T.P.'s Weekly" makes a practical and generous offer. The writer is, he says, in the Royal Army Medical Corps, has, as part of his training, attended several courses of nursing lectures, and was at one time a medical student. He says: "I wish to place my services and such knowledge of nursing as I possess (without remuneration) at the disposal of any young man living alone in London, who may be unwell at any time and need slight nursing attention." He adds, however, that there must be a limit to the distance from his residence in the Edgware Road which he would go to attend to anyone. We know nothing of the gentleman in question, but we think that his offer is one which others with some knowledge of nursing might be inclined to imitate. Nor need they offer gratuitous attendance. A young man in lodgings, ill enough to need something more than the casual attentions of a landlady, but unable to afford the cost of a nurse, and unable to accommodate her even if he can, would be glad of a visit from some one with a certain amount of skill in nursing, and in many cases would prefer someone of his own sex. Members of the Royal Army Medical Corps might not be available for everyone, but except in really serious cases the work might be undertaken by old soldiers who have been hospital orderlies, and by those who have been invalid attendants and valets. It would lie with the doctor attending such a case to say whether the daily, or morning and evening, visit of someone who had tact, kindliness, and some skill in handling a sick person, was enough, or whether a fully trained nurse was wanted. But it is undeniable that many men suffer much discomfort, and may even find slight illnesses develop into serious ones for want of a little care at the beginning.

### The Attenuation of Syphilitic Virus.

In the course of his third Harben lecture, Professor Metchnikoff detailed an accidental demonstration of the attenuation of the virus of syphilis by passage through the Macacus rhesus monkey. One of his assistants who had been occupied in the investigation of experimental syphilis developed a suspicious ulcer on his lower lip. However, the ulcer healed rapidly and was soon forgotten. A month later a similar ulcer appeared upon the same lip, but without any accompanying involvement of the lymphatic glands. Although there was little reason to suspect the ulcer of being syphilitic it was decided to set the matter at rest by inoculating a Javanese macacus with some exudation withdrawn from the ulcer. To the surprise of everyone the inoculated monkey developed two typical primary lesions four weeks later, while examination of these chancres revealed the spirochæte of Schaudinn in large abundance. In consequence the assistant sought the advice of Fournier who denied the necessity for anti-syphilitic treatment. This advice was justified by the total absence of any further evidences of syphilitic infection. There seems to be no doubt that the assistant in question had never previously suffered from syphilis. Upon the strength of this evidence Professor Metchnikoff consented to the inoculation of a human subject with a virus attenuated by passage through the macacus monkey. A volunteer came forward in the person of an aged female who after a minute examination was considered to be free from any past taint of the disease. Inoculation was followed after twelve days' incubation, by the appearance of two reddish-brown pustules similar to those of secondary syphilis, but by no further evidences of the disease. This discovery, though in the highest degree interesting, does not, as the Professor observed, justify us in assuming that we have a satisfactory vaccine against syphilis. General paralysis and tabes dorsalis are both known to follow syphilitic infections of low virulence. Prophylactic vaccination for this disease is out of the question, except under very special circumstances.

